# Cook County Medical Society Proceedings for April

**Published:** 1853-04

**Authors:** 


*Cook County Medical Society Proceedings for April.*

At the regular monthly meeting of the Cook County Medical Society, held at the office of Dr. N. S. Davis, on Friday, April 5th, Dr. Herrick gave a brief history of two cases of puerperal convulsions, one in charge of a homeopathic practitioner in the city, the other in the practice of a regular physician in the country.

The subject of the first case was Mrs. P——, a young woman having a nervous sanguine temperament, in labor, at the end of the full term of gestation with a first child.

On being called to the case twenty-four hours after labor pains had commenced, the practitioner in attendance stated that the labor had progressed naturally for twelve hours, or up to the time at which convulsions commenced. After this the efforts at expulsion, though at times vigorous, were ineffectual during the twelve hours following; at the termination of which period, the reporter being called, found the patient violently convulsed at short intervals, insensible, breathing stertuous, conjunctiva much injected and pupils dilated.

On examination it was found that the head of the child, with the occiput towards the right acetabulum, had passed below the upper strait into the cavity of a well formed pelvis. The soft parts being fully relaxed, no obstacle or objection existed to an immediate resort to instruments for the purpose of effecting what would have been accomplished twelve hours before, had the patient been under the care of any judicious and skilful obstetrician, immediate delivery, with the forceps, the fœtus apparently the principal if not the only exciting cause of the convulsions.

The convulsions ceased in this case with the delivery of the child, but their continuance of a violent character for so long a time, had so impaired the vital powers, that death from cerebral congestion followed in forty-eight hours after.

It is hoped that the melancholy termination of this case may serve as a salutary warning to the public, not to trust to sugar pills when life is at stake, and as additional evidence as it is to physicians, of the importance of early delivery by instruments, or otherwise, in all cases of puerperal convulsions.

The other case to which Dr. Herrick called the attention of the Society, was one to which he was called on Sunday last (the 3d inst.) the patient being as in the case previously cited, a young woman in labor with her first child, at the termination of a full term of gestation. In this case the labor progressed naturally for several hours, and until the head of the child began to press upon perineum, when convulsions commenced, at first sight, but more and more severe and at shorter intervals, up to a time twelve hours after their first appearance.

In this case the unwarrantable delay in delivery was for want of instruments, the practitioner in attendance being unprovided, and obliged to send 22 miles for the reporter and his forceps.

Dr. Herrick, on his arrival, found the patient insensible; convulsions recurring as often as every two or three hours; pulse full; respiration but slightly impaired, with eyes indicating no serious cerebral congestion.

Delivery with forceps was effected in a very few minutes, the uterus contracting sufficiently to prevent hemorrhage and favor the removal of the placenta. No convulsions or other unfavorable symptoms occurred during the two hours following; the indications, however, were so favorable, that there is but little doubt that the case wlll terminate favorably.

Dr. Palmer stated that it was his opinion, based upon experience and observation, that the treatment of puerperal convulsions with opium, or other strong anodynes, was not only inefficient but deleterious; has been called in consultation to several cases, in which convulsions came on after its administration by the practitioner in attendance; would, therefore, as a general rule, not give opium during labor: believes it should always be discarded when there is pain in the head and flushed face; may be given in cases in which the labor is irregular and slow, without any tendency to cerebral congestion; believes pueperal convulsions are frequently caused by the pains leaving the uterus and affecting the head or other parts; such cases are treated most effectually by bleeding and ergot.

Dr. Davis stated that he had been led to avoid the use of opium in labor, because of the effects which he has seen produced by it in several cases under the care of practitioners who were in the habit of using it freely in labor; believes he has seen four or five cases in which the pains were arrested permanently by its use; in practice has adopted the rule, never to give opium when the pains are efficient and regular, and the soft parts dilated or dilatable.

Dr. Miller remarked, that what had been said recalled to his mind a case which once came under his observation, in which convulsions continued obstinately after delivery, and in which there was little or no tendency to healthy uterine contraction. In this case morphine had been given without effect, but after the administration of ergot, pains came on, expelling a large amount of coagulated blood, after which the convulsions ceased.

For want of space in this number, we are obliged to reserve the remarks of other members of the Society, upon this subject for our next.

*Cook County Medical Society.*

